# Identification of Hub Genes Involved in Tubulointerstitial Injury in Diabetic Nephropathy by Bioinformatics Analysis and Experiment Verification

**DOI:** 10.1155/2022/7907708

**Published:** 2022-08-12

**Authors:** Jiayi Yang, Li Peng, Yuqiu Tian, Wenbin Tang, Linlin Peng, Jianping Ning, Dongjie Li, Yun Peng

**Affiliations:** ^1^Department of Geriatrics, Xiangya Hospital, Central South University, Changsha, Hunan 410008, China; ^2^National Clinical Research Center for Geriatric Disorders, Xiangya Hospital, Central South University, Changsha, Hunan 410008, China; ^3^Department of Ophthalmology, Central South University Xiangya School of Medicine Affiliated Haikou Hospital, Haikou, Hainan 570208, China; ^4^Department of Ophthalmology, The Second Xiangya Hospital, Central South University, Changsha, Hunan 410000, China; ^5^Department of Infectious Disease, Zhuzhou Central Hospital, Zhuzhou, Hunan 412000, China; ^6^Department of Nephrology, Xiangya Hospital, Central South University, Changsha, Hunan 410008, China; ^7^Health Management Center, Xiangya Hospital, Central South University, Changsha, Hunan 410008, China; ^8^Teaching and Research Section of Clinical Nursing, Xiangya Hospital, Central South University, Changsha, Hunan 410008, China

## Abstract

Diabetic nephropathy (DN) is the most important cause of end-stage renal disease with a poorer prognosis and high economic burdens of medical treatments. It is of great research value and clinical significance to explore potential gene targets of renal tubulointerstitial lesions in DN. To properly identify key genes associated with tubulointerstitial injury of DN, we initially performed a weighted gene coexpression network analysis of the dataset to screen out two nonconserved gene modules (dark orange and dark red). The regulation of oxidative stress-induced intrinsic apoptotic signaling pathway, PI3K-Akt signaling pathway, p38MAPK cascade, and Th1 and Th2 cell differentiation were primarily included in Gene Ontology (GO) annotation and Kyoto Encyclopedia of Genes and Genomes (KEGG) pathways of these two modules. Next, 199 differentially expressed genes (DEGs) were identified via the limma package. Then, the GO annotation and KEGG pathways of the DEGs were primarily enriched in extracellular matrix (ECM) organization, epithelial cell migration, cell adhesion molecules (CAMs), NF-kappa B signaling pathway, and ECM-receptor interaction. Gene set enrichment analysis showed that in the DN group, the interaction of ECM-receptor, CAMs, the interaction of cytokine-cytokine receptor, and complement and coagulation cascade pathways were significantly activated. Eleven key genes, including ALB, ANXA1, ANXA2, C3, CCL2, CLU, EGF, FOS, PLG, TIMP1, and VCAM1, were selected by constructing a protein-protein interaction network, and expression validation, ECM-related pathways, and glomerular filtration rate correlation analysis were performed in the validated dataset. The upregulated expression of hub genes ANXA2 and FOS was verified by real-time quantitative PCR in HK-2 cells treated with high glucose. This study revealed potential regulatory mechanisms of renal tubulointerstitial damage and highlighted the crucial role of extracellular matrix in DN, which may promote the identification of new biomarkers and therapeutic targets.

## 1. Introduction

Diabetic nephropathy (DN) is a principal microvascular complication with diabetes, which also is one of the most important causes of end-stage renal disease (ESRD). It imposes substantial personal and economic burdens on society and greatly reduces patients' quality of life [1, 2]. In recent decades, the prevalence of DN among diabetic patients has consistently been over 20%, while the prevalence of reduced glomerular filtration rate (GFR) has been increasing annually. The number of adults with diabetes is expected to increase to 642 million by 2040, 30%–40% of whom will develop DN [3]. DN is dominated by persistent albuminuria and/or progressive decrease in GFR and develops insidiously and slowly, eventually leading to ESRD. Because of the complex mechanism of DN, its prevention and treatment strategies have been the research hotspots at home and abroad, although no breakthrough progress has yet been made. Current comprehensive treatments of DN include lifestyle guidance, glycemic and lipid control, blood pressure and proteinuria management, and renal replacement therapy [4]. Although strict blood glucose and blood pressure control can effectively delay the progression of DN, many patients will still develop ESRD. Therefore, early diagnosis and effective treatment are essential for the prognosis of DN.

Renal tubulointerstitial injury is the primary cause of early DN, and its severity is closely associated with renal impairment in DN, which determines the long-term prognosis of the disease [5]. In the pathogenesis of DN, various mechanisms such as metabolic abnormalities, inflammation, oxidative stress, and hemodynamic changes can mediate renal tubulointerstitial lesions including inflammation and fibrosis, which play key roles in the progression of DN. The mechanisms of tubulointerstitial lesions in DN have been studied in great depth, resulting in the identification of many genes that have been found to constitute a complex pathway network, and new strategies for the treatment of DN have been obtained, therefore. For example, Sirtuin 1 was reported to be a new therapeutic target for patients with DN, due to it reducing inflammation in DN by inhibiting NF-*κ*B acetylation and activity [6]. Additionally, kidney-targeting Smad7 gene transfer can block transforming growth factor- (TGF-) *β*/Smad signaling to inhibit tubulointerstitial fibrosis in DN [7]. Therefore, an in-depth investigation of the genetic targets of tubulointerstitial renal lesions in DN has significant research value and clinical significance. In recent years, bioinformatics analysis has promoted new strategies for DN study.

In the present study, we used the GSE104954 dataset downloaded from the Gene Expression Omnibus (GEO) database by performing weighted gene coexpression network analysis (WGCNA) to find out highly coregulated and DN closely associated gene modules for further analysis of Gene Ontology (GO) and Kyoto Encyclopedia of Genes and Genomes (KEGG). Moreover, the differentially expressed genes (DEGs) were screened from the dataset and analyzed by GO and KEGG. Then, the gene set enrichment analysis (GSEA) was performed and a protein-protein interaction (PPI) network was constructed. Finally, 11 key genes were selected for further study. Validation of the expression, ECM-related pathways, and GFR correlation analysis of the 11 key genes was performed via the GSE30529 dataset, and experimental validation was performed using high glucose-treated HK-2 cells. This study revealed potential regulatory mechanisms underlying tubulointerstitial injury in DN and may promote the identification of novel biomarkers and therapeutic targets.

## 2. Results

### 2.1. Construction of a Coexpression Network

**Figure 1:**
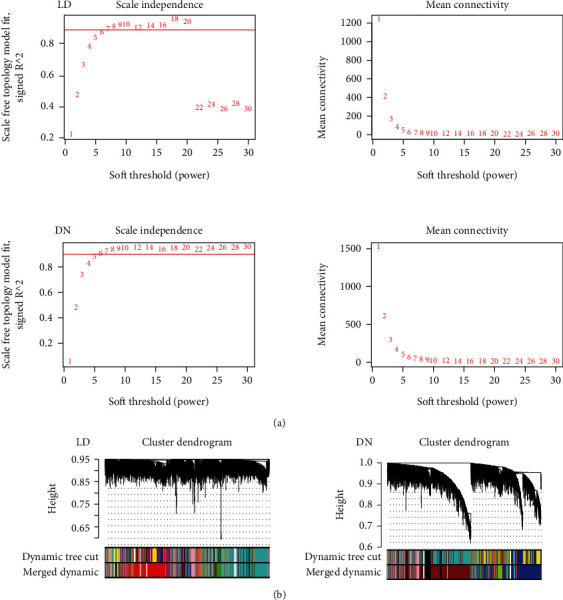
Gene modules in WGCNA analysis. (a) The soft power selection result of the DN and LD groups. (b) Module assignment in hierarchical clustered genes in the DN and LD groups. Genes within different modules are labeled with different colors according to WGCNA's conventions. DN: diabetic nephropathy; LD: living donors; WGCNA: weighted gene coexpression network analysis.

A coexpression network was constructed at the transcriptional level using the WGCNA method. We then compared differences between the specific networks of the DN and LD groups. The soft power selection results of the two groups are shown in Figure 1(a). First, after clustering, one sample, GSM2811043, was removed from the LD group; the final number of renal tubule samples in the GSE104954 database was 17 in the DN group and 20 in the LD group; their detailed information is listed in Supplementary Table S1. We continued to cluster and merge the modules. We then set the height of the clustering tree to 0.3, calculated the distance matrix using the Pearson correlation coefficient between the modules, and merged the modules. Modules with correlation over 0.7 were merged into a new module. The clustering diagram of the DN and LD groups is shown in Figure 1(b).

### 2.2. GO and KEGG Pathway Analyses of Characteristic Modules

**Figure 2:**
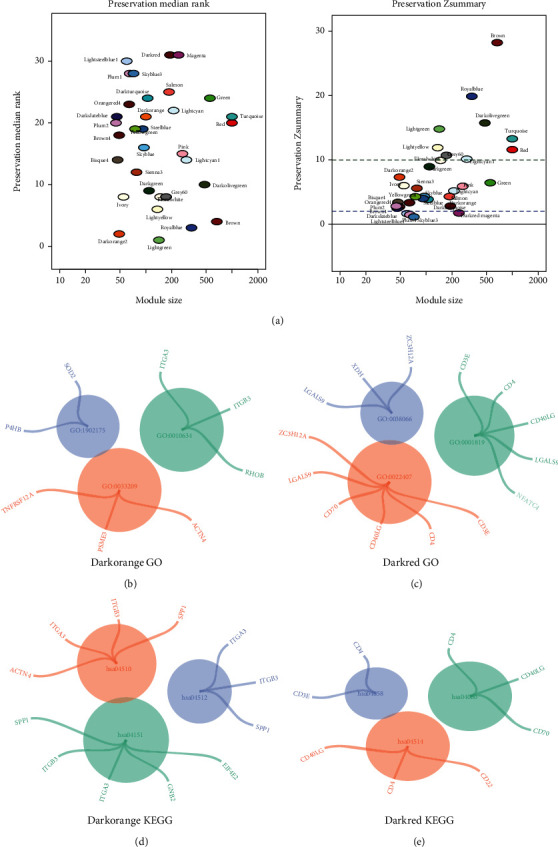
Characterizations of gene modules in WGCNA. (a) Preservation median rank and *Z* summary score of all modules were presented. The lowest *Z* summary statistics indicates nonconserved modules. The top two gene modules most significantly related with DN (dark orange, dark red) were selected for further analysis. The blue and green dotted lines indicate the thresholds *Z* = 2 and *Z* = 10, respectively. (b, c) GO enrichment results for dark orange and dark red modules. (d, e) KEGG pathway enrichment results for dark orange and dark red modules. GO: Gene Ontology; KEGG: Kyoto Encyclopedia of Genes and Genomes.

To verify the robustness of WGCNA, we analyzed the conservation of the modules. The preservation median rank and *Z* summary score of each module are detailed in Figure 2(a). Accordingly, we selected two characteristic modules (nonconserved modules with the lowest *Z* summary) of DN (dark orange and dark red) for GO analysis. The most important biological functions in the dark orange module included regulation of oxidative stress-induced intrinsic apoptotic signaling pathway, Notch receptor processing, and tumor necrosis factor-mediated signaling pathway (Figure 2(b)); the most important biological functions in the dark red module were primarily enriched in the p38MAPK cascade, regulation of cell-cell adhesion, and positive regulation of cytokine production (Figure 2(c)). Detailed information about the functional enrichment could be found in Supplementary Tables S2 and S3. Analysis of the KEGG pathways of the two modules showed that the dark orange module was primarily enriched in extracellular matrix- (ECM-) receptor interaction, focal adhesion, and PI3K-Akt signaling pathway (Figure 2(d)), whereas the main pathways in the dark red module included cell adhesion molecules (CAMs), Th1 and Th2 cell differentiation, and cytokine-cytokine receptor interaction (Figure 2(e)). Significant enriched pathways are listed in Supplementary Tables S4 and S5.

### 2.3. Identification and Enrichment Analyses of DEGs Associated with Tubulointerstitial Injury in DN

**Figure 3:**
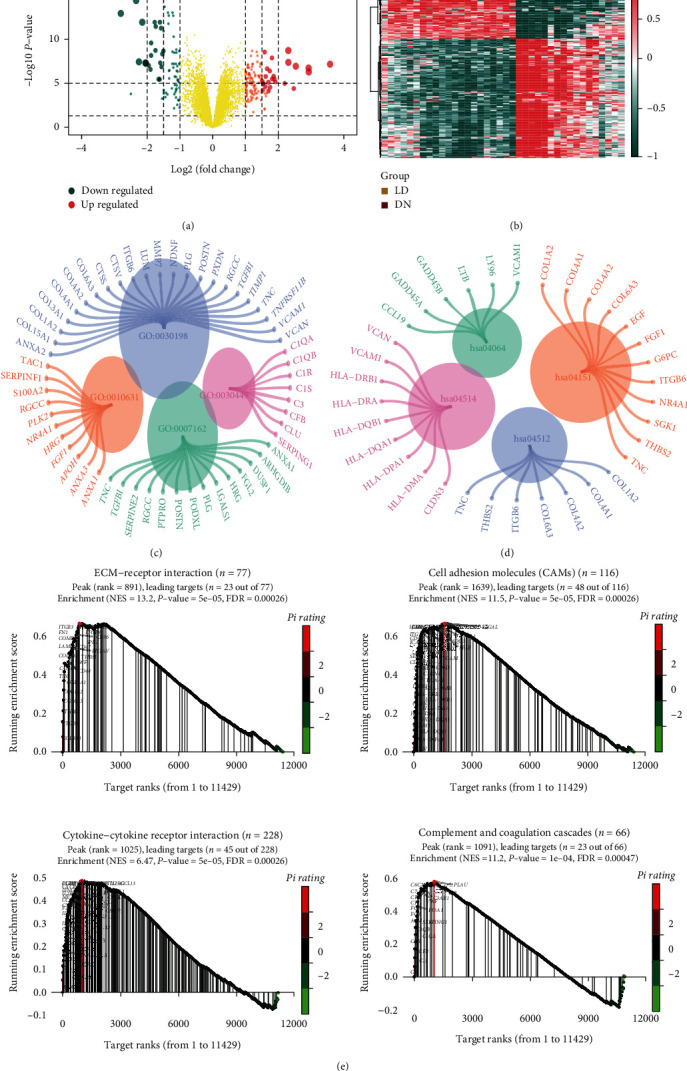
Identification and enrichment analyses of DEGs related to tubulointerstitial injury in DN. (a) Volcano plot of differential expressed genes (DEGs) between DN and LD tubule samples. The *x*-axis corresponds to log_2_ transformed fold change, and the *y*-axis corresponds to –log_10_ transformed *P* value. (b) Heat map of DEGs among 2 groups. (c, d) GO and KEGG pathway enrichment results for DEGs. (e) GSEA between the DN and LD groups. DEG: differentially expressed gene; GSEA: gene set enrichment analysis.

After merging and normalizing the microarray data, 199 DEGs closely related to DN were identified via the R package limma (*P* < 0.05 and ∣logFC | ≥1); the volcano plot and heat map of DEGs are shown in Figures 3(a) and 3(b), respectively. Next, we obtained the GO and KEGG pathway enrichment for all DEGs. The DEGs were significantly enriched in GO functions, including extracellular matrix organization, epithelial cell migration, negative cell adhesion regulation, and complement activation regulation (Figure 3(c)). Furthermore, the DEGs were significantly enriched with KEGG pathways, including PI3K-Akt signaling pathway, cell adhesion molecules (CAMs), ECM-receptor interaction, and NF-kappa B signaling pathway (Figure 3(d)). Significant enriched terms or pathways are listed in Supplementary Tables S6 and S7. GSEA was performed to identify the gene sets that were statistically different between the normal controls and DN group. The results illustrated that the gene sets and DN group were positively correlated which were significantly enriched in interaction of ECM-receptor CAMs, interaction of cytokine-cytokine receptor, and complement and coagulation cascades (Figure 3(e)).

### 2.4. Analysis of PPI Network and Recognition of Key Genes

**Figure 4:**
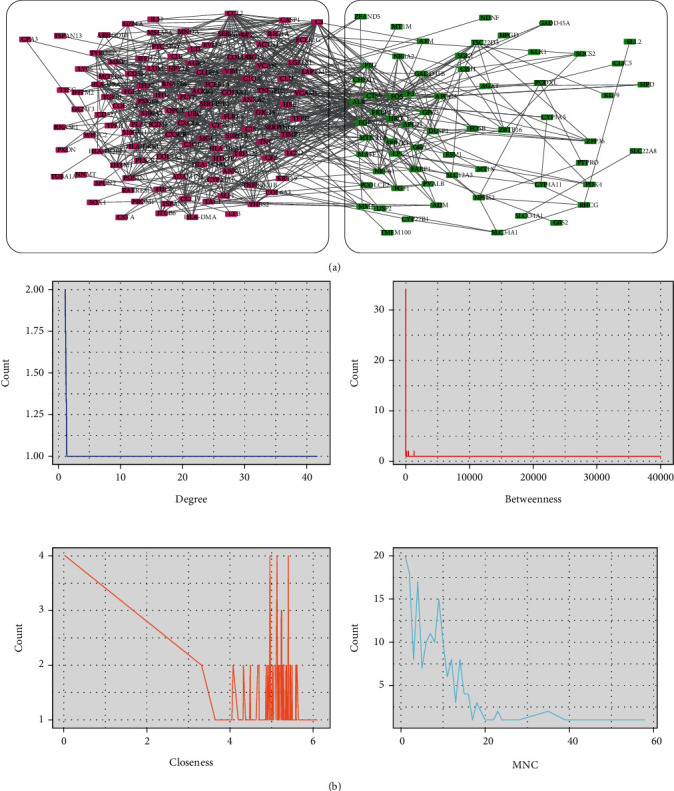
PPI network analysis and key genes' recognition. (a) PPI network for DEGs. Red rectangle node: upregulated genes; green rectangle node: downregulated genes. (b) Key genes were screened using Cytoscape cytoHubba plugin based on the four methods, Degree, Betweenness, Closeness, and MNC. PPI: protein–protein interaction.

**Figure 5:**
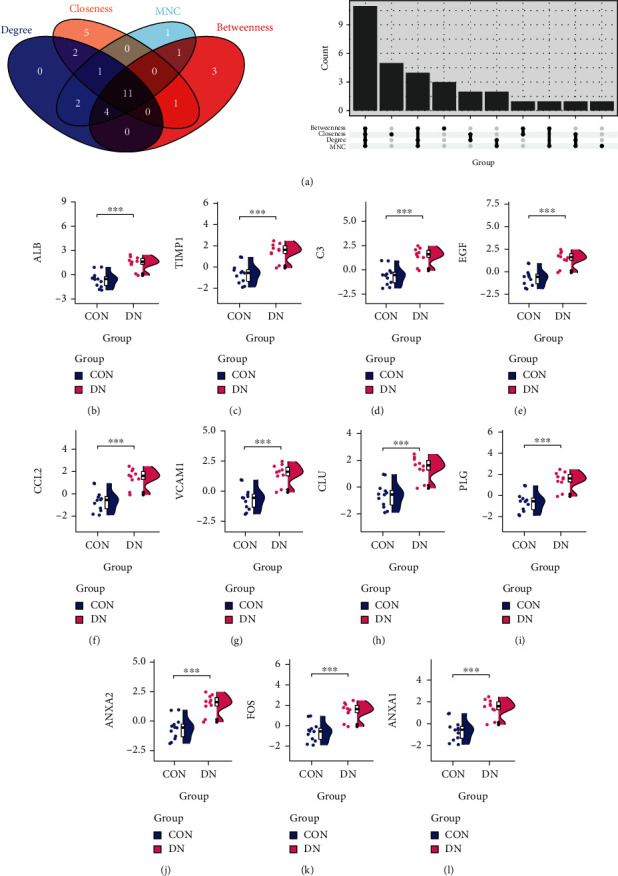
Screening and validation of key genes. (a) 11 key genes were selected from the intersected top 20 genes in the above four screening methods. Validation of expression levels of hub genes from the GSE30529 dataset. 11 key genes showed the similar upregulation trend: (b) ALB, (c) TIMP1, (d) C3, (e) EGF, (f) CCL2, (g) VCAM1, (h) CLU, (i) PLG, (j) ANXA2, (k) FOS, and (l) ANXA1.

The PPI network of DEGs was constructed by using STRING and then was visualized using Cytoscape software (Figure 4(a)). Key genes were screened using the Cytoscape cytoHubba plugin based on the four methods, Degree, Betweenness, Closeness, and MNC (Figure 4(b)), and the top 20 ranked genes were intersected, resulting in 11 key genes: ALB, ANXA1, ANXA2, C3, CCL2, CLU, EGF, FOS, PLG, TIMP1, and VCAM1 (Figure 5(a)). Exact value of the Degree, Betweenness, Closeness, and MNC for hub genes could be found in Supplementary Table S8.

### 2.5. Dataset Validation, ECM-Related Pathway, and GFR Correlation Analysis

**Figure 6:**
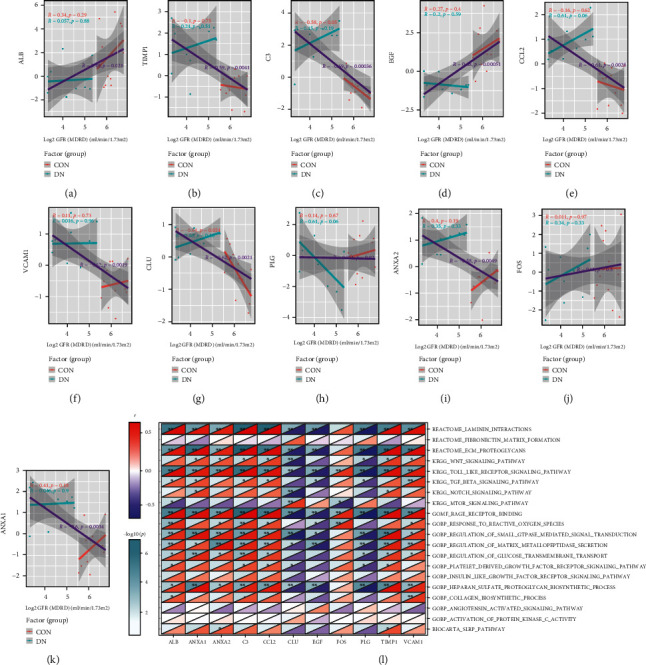
Correlation between ECM-related pathways, GFR, and key genes in DN. The expression of (b) TIMP1, (c) C3, (e) CCL2, (f) VCAM1, (g) CLU, (i) ANXA2, and (k) ANXA1 was reversely correlated with GFR. (l) Many key genes show significant correlation with the ECM-related pathways.

We validated the above 11 key genes in the GSE30529 dataset and found that they demonstrated similar patterns of upregulated expression (Figures 5(b)–5(l)). The expression of TIMP1, C3, CCL2, VCAM1, CLU, ANXA2, and ANXA1 was reversely correlated with GFR (Figures 6(a)–6(k)). We also investigated the correlation between ECM-related pathways and the 11 key genes, and we found that many of the genes showed significant correlation with these pathways (Figure 6(l)).

### 2.6. Validation of the Key Genes

**Figure 7:**
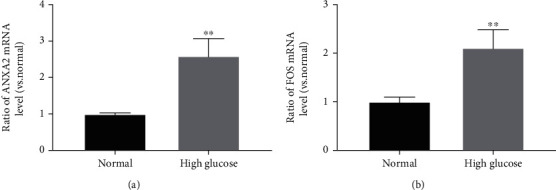
Validation of the expression level of three key genes using RT-qPCR in HK-2 cells under high-glucose stimulation: (a) ANXA2 and (b) FOS. ^∗∗^*P* < 0.01; unpaired Student's *t*-test.

The RT-qPCR showed that ANXA2 and FOS were upregulated in HK-2 cells under a high-glucose environment, and the differences were statistically significant compared with the control group (Figures 7(a) and 7(b)).

## 3. Discussion

DN has become the primary cause of dialysis treatment in chronic kidney disease patients and is an important cause of death from diabetes. The pathogenesis of DN is complex and remains unclear to a large extent. Previous studies on DN focused primarily on glomerular lesions. Recently, some studies focused on tubulointerstitial pathogenesis in DN to identify important genes associated with tubulointerstitial damage and to provide novel insights into its pathogenesis by emerging bioinformatics developments. For example, by analyzing DEGs in DN, Zeng et al. screened key genes to investigate their associations with clinical features (i.e., GFR, creatinine, and proteinuria). They identified drugs that prevent diabetic tubular interstitial injury and proposed many key genes involved in diabetic tubulointerstitial damage [8]. Song et al. identified VAV1, LCK, and Plk as reliable biomarkers of DN that can be used as indicators of development of DN in clinical management [9]. Furthermore, EST1 was found to be an important transcription factor in the development of DN, promoting the expression of integrin subunit beta 2, and may be a drug target in DN therapy [10]. Although some other genes have been reported in DN, the regulatory network of these key genes and related signaling pathways remain unclear.

In this study, we first identified the unconserved modules by WGCNA analyses. The GO or KEGG annotation of dark orange module showed their association with oxidative stress-induced apoptosis pathway, tumor necrosis factor- (TNF-) mediated signaling pathway, and Notch receptor processing, which is consistent with previous studies' results. Oxidative stress is an important process in DN pathogenesis. Meanwhile, it can mediate apoptosis in proximal tubular epithelial cells by affecting the expression of multiple caspases [11, 12]. TNF-*α* is a prime inducer and driver of renal microinflammation and is centrally acting in the proinflammatory molecular network of DN. In DN, TNF-*α* mediates activation of the protein kinase/phosphoinositide 3-kinase pathway or NADPH oxidase, which eventually produces reactive oxygen species that cause cellular damage. TNF-*α* also decreases nephrin expression and reduces Akt activity by mediating PI3K-Akt pathway activation, leading to reduced cell survival [13]. Notably, as a representative receptor involved in the Notch protein family, activation of Notch-1 is closely related to the degree of podocyte injury induced by high glucose [14]. The GO annotations of the dark red module indicated its correlation with p38MAPK cascade, regulation of cell-cell adhesion, and cytokine production with positive regulation. The inflammatory response in diabetes is a key factor that can contribute to the activation of the p38MAPK signaling pathway, thereby inducing activation of downstream inflammatory cells and promoting the expression of inflammatory factors further to aggravate renal damage [15, 16]. In DN, many signaling pathways and molecular networks in vivo induce mesenchymal cell properties in interepithelial cells by inhibiting their expression of E-cadherin and decreasing their adhesiveness, resulting in renal fibrosis [17]. KEGG enrichment analysis of the dark orange module presented the involvement of the interaction of ECM-receptor, focal adhesion, and the PI3K-Akt signaling pathway, demonstrating the ECM changes in DN, and this was further validated by the correlation between ECM-related pathways and key genes, whereas the main pathways enriched for genes in the dark red module also included CAMs, Th1 and Th2 cell differentiation, and interaction of cytokine-cytokine receptor. This suggests a key role for the PI3K-Akt signaling pathway and T cell immune responses in DN. The PI3K-Akt signaling pathway has been implicated as an important pathogenic mechanism in DN [18, 19]. An increasing number of studies have also confirmed that Th1 and Th2 cells are involved in the development of DN [20, 21]. Similarly, we observed enrichment of immunological and ECM-related pathways based on the DEGs between control and DN samples, including ECM organization, epithelial cell migration, cell adhesion with negative regulation, and regulation of complement activation. Tubular epithelial myofibroblast transdifferentiation can be observed in DN, and epithelial cell transdifferentiation results in loss of adhesion, increased migration, and abnormal accumulation because of secreted ECM, thereby promoting the progression of kidney interstitial fibrosis [22, 23]. Furthermore, evidence has revealed the important role of complement activation in tubulointerstitial injury in DN [24, 25]. Additionally, NF-kappa B signaling was enriched, and it is a key mechanism underlying the inflammatory response in DN [26]. All the results suggested the engagement of ECM-related pathological mechanisms in DN development and their potential interaction with immunocytes, highlighting the critical role of immune-stromal interplay.

We identified 11 key genes (ALB, ANXA1, ANXA2, C3, CCL2, CLU, EGF, FOS, PLG, TIMP1, and VCAM1) and validated their expression and correlation with ECM-related pathways, GFR. Among them, ANXA2 and FOS deserve further investigation due to their biological importance and unclear role in the renal tubulointerstitial injury of DN. Therefore, we confirmed ANXA2's and FOS's upregulation in HK-2 cells under a high-glucose environment by RT-qPCR. ANXA2, an important member of the annexin family, is a calcium-dependent phospholipid-binding protein with various biological functions, including cell proliferation, apoptosis, migration, invasion, and adhesion regulation. Previous studies on the ANXA2 mechanism mainly focused on tumor diseases [27]. In recent years, only a few studies initially suggested that ANXA2 may be related to DN. ANXA2 was found to affect the occurrence of DN [28], and the protein levels of ANXA2 and antiproliferative molecules were upregulated in the glomeruli of diabetic KKAy mice [29]. ANXA2 also played a role in the miR-151-3p/ANXA2 axis influenced by interference of Hsa_circ_0003928, which alleviated high glucose-induced HK-2 cell apoptosis and inflammation [30]. However, no studies report the specific mechanisms and pathways of ANXA2 in the occurrence and development of DN, especially its role in diabetic tubulointerstitial lesions. Hence, we are committed to ANXA2 in tubulointerstitial injury in DN, an area with no known evidence. FOS is a nuclear phosphoprotein and encodes the nuclear oncoprotein c-Fos. As a class of nuclear protein transcription factor, FOS has important roles in regulating cell growth, division, proliferation, differentiation, and programmed death. In glomerular mesangial cells under a high-glucose environment, c-Fos protein expression is significantly upregulated and the phosphorylation of c-Fos (ser32) is increased, which promotes the downstream gene expression, leading to DN development [31, 32]. However, the specific mechanism of its effects is still unclear in DN and was not reported in the tubulointerstitial injury of DN. Discovering the mechanisms of ANXA2 and FOS in DN will improve the understanding of DN pathogenesis and benefit novel DN therapeutic strategy development.

In summary, this study identified and selected underlying genes that may play a significant role in the pathogenesis of tubulointerstitial injury in DN. Meanwhile, it highlighted the importance of tubulointerstitial injury in DN, especially in providing more in-depth research on the molecular level. However, we acknowledge that our study had a few potential limitations that must be considered. First, the original microarray data lacked sufficient clinical data and experimental results. Second, we only used renal tubular epithelial cell models to verify our results, while no animal experiments were conducted. Further studies at multiple research levels are necessary to illustrate additional insights into the diagnosis and progression of diabetic tubular interstitial lesions in order to improve the management of this disease.

## 4. Conclusion

In conclusion, we identified unconserved gene modules between DN and LD and noticed that they were enriched in immune and ECM-related pathways. Subsequently, we screened out 11 key genes involved in tubulointerstitial injury in DN via bioinformatics analysis and validated that they were upregulated and correlated with ECM-related pathways and GFR in DN. Further, we confirmed the upregulation of ANXA2 and FOS in DN via RT-qPCR experiments. Identification of these genes may shed light on the ECM-related mechanisms of DN and benefit effective therapeutic strategy development.

## 5. Methods

### 5.1. Microarray Data Acquisition

**Figure 8:**
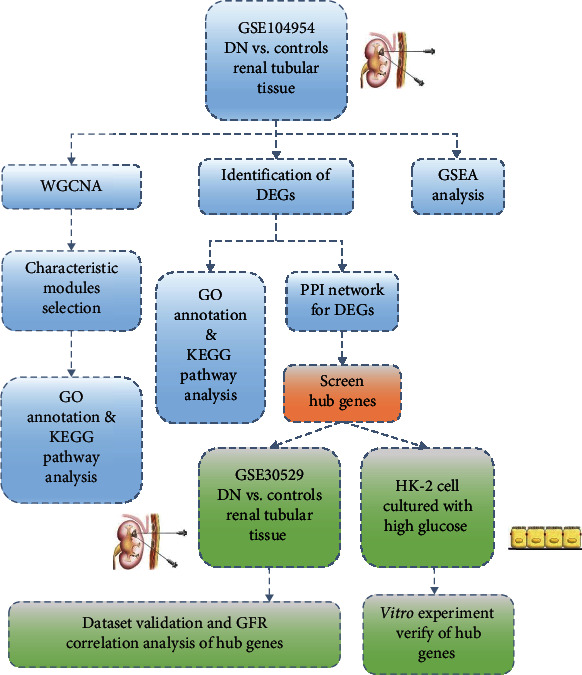
The flowchart designed for the study.

The GSE104954 and GSE30529 datasets were selected from the Gene Expression Omnibus (GEO) database for this study. GSE104954 is based on GPL22945 (Affymetrix Human Genome U133 Plus 2.0 Array) and GPL24120 (Affymetrix Human Genome U133A Array) platforms. They include 17 renal tubulointerstitial tissue samples of the patients with DN and 21 normal control sample. GSE30529 was performed with the GPL571 Array platform (Affymetrix Human Genome U133 2.0), which contains 10 renal tubular tissue samples of patients with DN and 12 normal control samples. The GSE30529 dataset was used for gene expression validation and clinical characterization. ECM-related gene sets were downloaded from GSEA. The workflow designed for the study is shown in Figure 8.

### 5.2. Screening of DEGs

Data preprocessing consisted of conversion from gene probes into gene symbols, consolidation of data, and batch normalization. The gene probes with no gene symbols or genes with multiple probes were removed or taken the maximum probe, respectively. The merged data were preprocessed via the R package sva [33] (version 3.5.3) (Broad Institute, Inc., Massachusetts Institute of Technology, and California, USA) to eliminate batch effects. Post batch normalization, DEGs were identified using the R package limma [34] with adjusted *P* value < 0.05 and ∣logFC | ≥1 in renal tubulointerstitial tissues from patients of DN and healthy controls. Heat maps of DEGs were plotted using the Pheatmap R package.

### 5.3. Weighted Gene Coexpression Network Analysis (WGCNA)

WGCNA is a biological method for identifying genes with similar expression patterns among different samples. It is used to identify coexpressed gene modules by sample clustering and explores the association between gene networks and clinical phenotypes. After preprocessing with raw data, a WGCNA was performed via the WGCNA package in R [35] to identify significant gene modules for analysis. To summarize, the Pearson correlation coefficients of the chosen genes were calculated in a pairwise fashion, and the similarity matrix (*S*_*ij*_) was obtained. The soft thresholding power was set to “7” and “8” for the DN and living donor (LD) groups, respectively. Based on the scale-free topology, the soft threshold “*β*” was used as the weight coefficient to achieve scale-free of the network (*R*^2^ = 0.9). The matrix was transformed to an adjacency matrix (*a*_*ij*_) via a power function. Average linkage hierarchical clustering was conducted, and closely associated genes of the classification modules were then created. The different functions were symbolized as tomtype according to the topological overlap matrix, and the network was interconnected by calculating the topological overlap. Based on their linkage strength, genes were constructed on a 1-TOM basis, and genes were grouped according to the average hierarchical clustering, which is measured by the hclust function. A module represented a set of extremely coexpressed genes, generally consisting of over 30 genes. Then, genes that were not assigned to a specific module were designated as gray. The module preservation function was used to analyze module conservation between the DN and LD group networks, and nonconserved DN-related modules (those with the lowest *Z* summary) were selected for subsequent analysis by combining both preservations median rank and *Z* summary statistics.

### 5.4. Pathway Analysis

GO is a bioinformatics initiative to annotate genes and proteins to determine their characteristic biological properties, including biological processes, cellular components, and molecular functions. The KEGG pathway database is a resource for understanding the high-level function and utility of biological systems, which includes various biochemical pathways. In this study, the cluster profiler package [36] was used for GO and KEGG analyses.

### 5.5. Gene Set Enrichment Analysis (GSEA)

GSEA (Broad Institute, Inc., Massachusetts Institute of Technology, California, USA) applies computational methods to help determine significant differences between gene sets. GSEA was performed using the R package Pi. ∣NES | >1, *P* < 0.05, and *q* < 0.25 were considered statistically significant.

### 5.6. Construction of PPI Network and Identification of the Key Genes

PPI networks of DEGs were established using search tools for retrieval of interacting genes (STRING v10; http://string-db.org [37]) and were visualized using Cytoscape software [38]. Next, key genes were screened in Cytoscape cytoHubba [39], and key genes were obtained using four criteria including Degree, Betweenness, Closeness, and MNC.

### 5.7. Validation of the Key Genes

#### 5.7.1. Cell Cultivation and High-Glucose Stimulation

HK-2 cells were maintained with DMEM/F12 medium containing 10% FBS in the conditions: 95% air and 5% CO_2_ at 37°C in an incubator, and passaged or preserved every 2–3 days. Cells were observed under microscopy and digested with 0.25% trypsin solution for passaging when they reached 80%–90% confluence. HK-2 cells were separated into the two groups: high-glucose group (DMEM/F12 medium supplemented with 30 mmol/L glucose) and normal group (DMEM/F12 medium supplemented with 5.5 mmol/L glucose).

#### 5.7.2. Microarray Data Acquisition

Total RNA was extracted using TRIzol reagent (Invitrogen, Carlsbad, CA) based on the manufacturer's instructions. Then, total RNA was reverse transcribed to cDNA using a MiScript reverse transcription kit (Qiagen, Hilden, Germany) to measure the expression of key genes. mRNA expression was measured based on the reverse transcription via a PrimeScript RT kit (Takara, Tokyo, Japan). Relative expression (with GAPDH as control) was measured using SYBR premix Ex Taq II (TaKaRa, Tokyo, Japan) with an Applied Biosystems 7500 system (Thermo Fisher Scientific, Waltham, MA, USA). Relative expression was calculated using the 2∆∆Cq method. Primer sequences are shown in Table 1.

### 5.8. Analyses of ECM-Related Pathway Enrichment

The ECM-related pathways in DN were proposed in a review [40], and we calculated the enrichment levels of the gene sets using ssGSEA of GSVA [41]. The correlation between ECM-related gene sets and the 11 key genes was further analyzed and visualized in a heat map.

### 5.9. Statistical Analysis

The normality of the variables was tested via the Shapiro–Wilk test. For normally distributed variables, differences between the two groups were compared by the unpaired Student's *t*-test. The correlation between ECM-related pathways, GFR, and hub gene expression was quantified using Spearman's correlation coefficient. Data were statistically analyzed using the R statistical analysis package (version 3.5.3). The values of *P* < 0.05 were regarded statistically significant comparison in this study.

## Figures and Tables

**Table 1:** Real-time primer sequences of HK-2 cell line primer sequences.

Gene	Sequences
Actin	Forward: ACCCTGAAGTACCCCATCGAG
	Reverse: AGCACAGCCTGGATAGCAAC
ANXA2	Forward: GCACGGCCCAGGTTATCTT
	Reverse: ATGTGTTCAACCAAGCGGGA
FOS	Forward: CCGAGCTGGTGCATTACAGA
	Reverse: CGCACAGATAAGGTCCTCCC

## Data Availability

The GEO datasets can be retrieved from “https://www.ncbi.nlm.nih.gov/gds/?term=,” and the GO and KEGG gene sets were downloaded from the Molecular Signature Database of GSEA (http://www.gsea-msigdb.org/gsea/msigdb/index.jsp). The R codes can be available from the corresponding authors.

## Supplementary Materials

Supplementary Materials Supplementary Table S1: detailed information about the final input samples of WGCNA. Supplementary Table S2: detailed information about the functional enrichment of the dark orange module. Supplementary Table S3: detailed information about the functional enrichment of the dark red module. Supplementary Table S4: detailed information about the significant enriched pathways of the dark orange module. Supplementary Table S5: detailed information about the significant enriched pathways of the dark. Supplementary Table S6: detailed information about the significant enriched terms of DEGs related to DN. Supplementary Table S7: detailed information about the significant enriched pathways of DEGs related to DN. Supplementary Table S8: exact value of the Degree, Betweenness, Closeness, and MNC for hub genes.
