# Supplementary material for: Identification of Hub Genes Involved in Tubulointerstitial Injury in Diabetic Nephropathy by Bioinformatics Analysis and Experiment Verification
Source: J Immunol Res. 2022 Aug 12;2022:7907708. doi: 10.1155/2022/7907708 (PMC9391162; doi:10.1155/2022/7907708)
Supplement: Supplementary Materials — Supplementary Table S1: detailed information about the final input samples of WGCNA. Supplementary Table S2: detailed information about the functional enrichment of the dark orange module. Supplementary Table S3: detailed information about the functional enrichment of the dark red module. Supplementary Table S4: detailed information about the significant enriched pathways of the dark orange module. Supplementary Table S5: detailed information about the significant enriched pathways of the dark. Supplementary Table S6: detailed information about the significant enriched terms of DEGs related to DN. Supplementary Table S7: detailed information about the significant enriched pathways of DEGs related to DN. Supplementary Table S8: exact value of the Degree, Betweenness, Closeness, and MNC for hub genes. [file 7907708.f1.docx]

Identification of hub genes involved in tubulointerstitial injury in diabetic nephropathy by bioinformatics analysis and experiment verifies

**Jiayi Yang^1,2^, Li Peng^3,4^, Yuqiu Tian^5^, Wenbin Tang^6,7^, Linlin Peng^1,2^, Jianping Ning^6^, Dongjie Li^1,2^, Yun Peng^1,2,8,*^**

^1^Department of Geriatrics, Xiangya Hospital, Central South University, Changsha, Hunan, 410008, China.

^2^National Clinical Research Center for Geriatric Disorders, Xiangya Hospital, Central South University, Changsha, Hunan, 410008, China.

^3^Department of Ophthalmology, Central South University Xiangya School of Medicine Affiliated Haikou Hospital, Haikou, Hainan, 570208, China.

^4^Department of Ophthalmology, The Second Xiangya Hospital, Central South University, Changsha, Hunan, 410000, China.

^5^Department of Infectious Disease, Zhuzhou Central Hospital, Zhuzhou, Hunan, 412000, China.

^6^Department of Nephrology, Xiangya Hospital, Central South University, Changsha, Hunan, 410008, China.

^7^Health Management Center, Xiangya Hospital, Central South University, Changsha, Hunan, 410008, China.

^8^Teaching and Research Section of Clinical Nursing, Xiangya Hospital, Central South University, Changsha, Hunan, 410008, China.

*Corresponding authors: Yun Peng ([xyyy56lnyxk@163.com](mailto:xyyy56lnyxk@163.com)).

| SampleID | title | geo_accession | characteristics_ch1 | characteristics_ch1.1 | scan_protocol | description | data_processing | platform_id | data_row_count |
| --- | --- | --- | --- | --- | --- | --- | --- | --- | --- |
| GSM2811029 | H7-Tub-DN1101 | GSM2811029 | tissue: Tubulointerstitium from kidney biopsy | diagnosis: Diabetic nephropathy | GeneChip arrays were scanned by GeneChip Scanner 3000 7G according to the Affymetrix Expression Analysis Technical Manual. | Gene expression from the tubulointerstitial compartment of human H7-Tub-DN1101 | Each batch and platform was processed individually with its corresponding BrainArray CDF and normalized by RMA. After individual batch and platform processing, samples were merged by common EntrezGeneIDs, and finally batch corrected using Combat. | GPL22945 | 12074 |
| GSM2811030 | H7-Tub-DN1108 | GSM2811030 | tissue: Tubulointerstitium from kidney biopsy | diagnosis: Diabetic nephropathy | GeneChip arrays were scanned by GeneChip Scanner 3000 7G according to the Affymetrix Expression Analysis Technical Manual. | Gene expression from the tubulointerstitial compartment of human H7-Tub-DN1108 | Each batch and platform was processed individually with its corresponding BrainArray CDF and normalized by RMA. After individual batch and platform processing, samples were merged by common EntrezGeneIDs, and finally batch corrected using Combat. | GPL22945 | 12074 |
| GSM2811031 | H7-Tub-DN1110 | GSM2811031 | tissue: Tubulointerstitium from kidney biopsy | diagnosis: Diabetic nephropathy | GeneChip arrays were scanned by GeneChip Scanner 3000 7G according to the Affymetrix Expression Analysis Technical Manual. | Gene expression from the tubulointerstitial compartment of human H7-Tub-DN1110 | Each batch and platform was processed individually with its corresponding BrainArray CDF and normalized by RMA. After individual batch and platform processing, samples were merged by common EntrezGeneIDs, and finally batch corrected using Combat. | GPL22945 | 12074 |
| GSM2811032 | H7-Tub-DN1114 | GSM2811032 | tissue: Tubulointerstitium from kidney biopsy | diagnosis: Diabetic nephropathy | GeneChip arrays were scanned by GeneChip Scanner 3000 7G according to the Affymetrix Expression Analysis Technical Manual. | Gene expression from the tubulointerstitial compartment of human H7-Tub-DN1114 | Each batch and platform was processed individually with its corresponding BrainArray CDF and normalized by RMA. After individual batch and platform processing, samples were merged by common EntrezGeneIDs, and finally batch corrected using Combat. | GPL22945 | 12074 |
| GSM2811033 | H7-Tub-DN1132 | GSM2811033 | tissue: Tubulointerstitium from kidney biopsy | diagnosis: Diabetic nephropathy | GeneChip arrays were scanned by GeneChip Scanner 3000 7G according to the Affymetrix Expression Analysis Technical Manual. | Gene expression from the tubulointerstitial compartment of human H7-Tub-DN1132 | Each batch and platform was processed individually with its corresponding BrainArray CDF and normalized by RMA. After individual batch and platform processing, samples were merged by common EntrezGeneIDs, and finally batch corrected using Combat. | GPL22945 | 12074 |
| GSM2811034 | H7-Tub-DN1133 | GSM2811034 | tissue: Tubulointerstitium from kidney biopsy | diagnosis: Diabetic nephropathy | GeneChip arrays were scanned by GeneChip Scanner 3000 7G according to the Affymetrix Expression Analysis Technical Manual. | Gene expression from the tubulointerstitial compartment of human H7-Tub-DN1133 | Each batch and platform was processed individually with its corresponding BrainArray CDF and normalized by RMA. After individual batch and platform processing, samples were merged by common EntrezGeneIDs, and finally batch corrected using Combat. | GPL22945 | 12074 |
| GSM2811035 | H7-Tub-DN1139 | GSM2811035 | tissue: Tubulointerstitium from kidney biopsy | diagnosis: Diabetic nephropathy | GeneChip arrays were scanned by GeneChip Scanner 3000 7G according to the Affymetrix Expression Analysis Technical Manual. | Gene expression from the tubulointerstitial compartment of human H7-Tub-DN1139 | Each batch and platform was processed individually with its corresponding BrainArray CDF and normalized by RMA. After individual batch and platform processing, samples were merged by common EntrezGeneIDs, and finally batch corrected using Combat. | GPL22945 | 12074 |
| GSM2811044 | H7-Tub-LD1165 | GSM2811044 | tissue: Tubulointerstitium from kidney biopsy |  | GeneChip arrays were scanned by GeneChip Scanner 3000 7G according to the Affymetrix Expression Analysis Technical Manual. | Gene expression from the tubulointerstitial compartment of human H7-Tub-LD1165 | Each batch and platform was processed individually with its corresponding BrainArray CDF and normalized by RMA. After individual batch and platform processing, samples were merged by common EntrezGeneIDs, and finally batch corrected using Combat. | GPL22945 | 12074 |
| GSM2811045 | H7-Tub-LD1166 | GSM2811045 | tissue: Tubulointerstitium from kidney biopsy |  | GeneChip arrays were scanned by GeneChip Scanner 3000 7G according to the Affymetrix Expression Analysis Technical Manual. | Gene expression from the tubulointerstitial compartment of human H7-Tub-LD1166 | Each batch and platform was processed individually with its corresponding BrainArray CDF and normalized by RMA. After individual batch and platform processing, samples were merged by common EntrezGeneIDs, and finally batch corrected using Combat. | GPL22945 | 12074 |
| GSM2811046 | H7-Tub-LD1168 | GSM2811046 | tissue: Tubulointerstitium from kidney biopsy |  | GeneChip arrays were scanned by GeneChip Scanner 3000 7G according to the Affymetrix Expression Analysis Technical Manual. | Gene expression from the tubulointerstitial compartment of human H7-Tub-LD1168 | Each batch and platform was processed individually with its corresponding BrainArray CDF and normalized by RMA. After individual batch and platform processing, samples were merged by common EntrezGeneIDs, and finally batch corrected using Combat. | GPL22945 | 12074 |
| GSM2811047 | H7-Tub-LD1169 | GSM2811047 | tissue: Tubulointerstitium from kidney biopsy |  | GeneChip arrays were scanned by GeneChip Scanner 3000 7G according to the Affymetrix Expression Analysis Technical Manual. | Gene expression from the tubulointerstitial compartment of human H7-Tub-LD1169 | Each batch and platform was processed individually with its corresponding BrainArray CDF and normalized by RMA. After individual batch and platform processing, samples were merged by common EntrezGeneIDs, and finally batch corrected using Combat. | GPL22945 | 12074 |
| GSM2811048 | H7-Tub-LD1170 | GSM2811048 | tissue: Tubulointerstitium from kidney biopsy |  | GeneChip arrays were scanned by GeneChip Scanner 3000 7G according to the Affymetrix Expression Analysis Technical Manual. | Gene expression from the tubulointerstitial compartment of human H7-Tub-LD1170 | Each batch and platform was processed individually with its corresponding BrainArray CDF and normalized by RMA. After individual batch and platform processing, samples were merged by common EntrezGeneIDs, and finally batch corrected using Combat. | GPL22945 | 12074 |
| GSM2811049 | H7-Tub-LD1171 | GSM2811049 | tissue: Tubulointerstitium from kidney biopsy |  | GeneChip arrays were scanned by GeneChip Scanner 3000 7G according to the Affymetrix Expression Analysis Technical Manual. | Gene expression from the tubulointerstitial compartment of human H7-Tub-LD1171 | Each batch and platform was processed individually with its corresponding BrainArray CDF and normalized by RMA. After individual batch and platform processing, samples were merged by common EntrezGeneIDs, and finally batch corrected using Combat. | GPL22945 | 12074 |
| GSM2811050 | H7-Tub-LD1172 | GSM2811050 | tissue: Tubulointerstitium from kidney biopsy |  | GeneChip arrays were scanned by GeneChip Scanner 3000 7G according to the Affymetrix Expression Analysis Technical Manual. | Gene expression from the tubulointerstitial compartment of human H7-Tub-LD1172 | Each batch and platform was processed individually with its corresponding BrainArray CDF and normalized by RMA. After individual batch and platform processing, samples were merged by common EntrezGeneIDs, and finally batch corrected using Combat. | GPL22945 | 12074 |
| GSM2811051 | H7-Tub-LD1173 | GSM2811051 | tissue: Tubulointerstitium from kidney biopsy |  | GeneChip arrays were scanned by GeneChip Scanner 3000 7G according to the Affymetrix Expression Analysis Technical Manual. | Gene expression from the tubulointerstitial compartment of human H7-Tub-LD1173 | Each batch and platform was processed individually with its corresponding BrainArray CDF and normalized by RMA. After individual batch and platform processing, samples were merged by common EntrezGeneIDs, and finally batch corrected using Combat. | GPL22945 | 12074 |
| GSM2811052 | H7-Tub-LD1174 | GSM2811052 | tissue: Tubulointerstitium from kidney biopsy |  | GeneChip arrays were scanned by GeneChip Scanner 3000 7G according to the Affymetrix Expression Analysis Technical Manual. | Gene expression from the tubulointerstitial compartment of human H7-Tub-LD1174 | Each batch and platform was processed individually with its corresponding BrainArray CDF and normalized by RMA. After individual batch and platform processing, samples were merged by common EntrezGeneIDs, and finally batch corrected using Combat. | GPL22945 | 12074 |
| GSM2811053 | H7-Tub-LD1175 | GSM2811053 | tissue: Tubulointerstitium from kidney biopsy |  | GeneChip arrays were scanned by GeneChip Scanner 3000 7G according to the Affymetrix Expression Analysis Technical Manual. | Gene expression from the tubulointerstitial compartment of human H7-Tub-LD1175 | Each batch and platform was processed individually with its corresponding BrainArray CDF and normalized by RMA. After individual batch and platform processing, samples were merged by common EntrezGeneIDs, and finally batch corrected using Combat. | GPL22945 | 12074 |
| GSM2811054 | H7-Tub-LD1177 | GSM2811054 | tissue: Tubulointerstitium from kidney biopsy |  | GeneChip arrays were scanned by GeneChip Scanner 3000 7G according to the Affymetrix Expression Analysis Technical Manual. | Gene expression from the tubulointerstitial compartment of human H7-Tub-LD1177 | Each batch and platform was processed individually with its corresponding BrainArray CDF and normalized by RMA. After individual batch and platform processing, samples were merged by common EntrezGeneIDs, and finally batch corrected using Combat. | GPL22945 | 12074 |
| GSM2811055 | H7-Tub-LD1178 | GSM2811055 | tissue: Tubulointerstitium from kidney biopsy |  | GeneChip arrays were scanned by GeneChip Scanner 3000 7G according to the Affymetrix Expression Analysis Technical Manual. | Gene expression from the tubulointerstitial compartment of human H7-Tub-LD1178 | Each batch and platform was processed individually with its corresponding BrainArray CDF and normalized by RMA. After individual batch and platform processing, samples were merged by common EntrezGeneIDs, and finally batch corrected using Combat. | GPL22945 | 12074 |
| GSM2811056 | H7-Tub-LD1179 | GSM2811056 | tissue: Tubulointerstitium from kidney biopsy |  | GeneChip arrays were scanned by GeneChip Scanner 3000 7G according to the Affymetrix Expression Analysis Technical Manual. | Gene expression from the tubulointerstitial compartment of human H7-Tub-LD1179 | Each batch and platform was processed individually with its corresponding BrainArray CDF and normalized by RMA. After individual batch and platform processing, samples were merged by common EntrezGeneIDs, and finally batch corrected using Combat. | GPL22945 | 12074 |
| GSM2811057 | H7-Tub-LD1180 | GSM2811057 | tissue: Tubulointerstitium from kidney biopsy |  | GeneChip arrays were scanned by GeneChip Scanner 3000 7G according to the Affymetrix Expression Analysis Technical Manual. | Gene expression from the tubulointerstitial compartment of human H7-Tub-LD1180 | Each batch and platform was processed individually with its corresponding BrainArray CDF and normalized by RMA. After individual batch and platform processing, samples were merged by common EntrezGeneIDs, and finally batch corrected using Combat. | GPL22945 | 12074 |
| GSM2811058 | H7-Tub-LD1182 | GSM2811058 | tissue: Tubulointerstitium from kidney biopsy |  | GeneChip arrays were scanned by GeneChip Scanner 3000 7G according to the Affymetrix Expression Analysis Technical Manual. | Gene expression from the tubulointerstitial compartment of human H7-Tub-LD1182 | Each batch and platform was processed individually with its corresponding BrainArray CDF and normalized by RMA. After individual batch and platform processing, samples were merged by common EntrezGeneIDs, and finally batch corrected using Combat. | GPL22945 | 12074 |
| GSM2811059 | H7-Tub-LD1183 | GSM2811059 | tissue: Tubulointerstitium from kidney biopsy |  | GeneChip arrays were scanned by GeneChip Scanner 3000 7G according to the Affymetrix Expression Analysis Technical Manual. | Gene expression from the tubulointerstitial compartment of human H7-Tub-LD1183 | Each batch and platform was processed individually with its corresponding BrainArray CDF and normalized by RMA. After individual batch and platform processing, samples were merged by common EntrezGeneIDs, and finally batch corrected using Combat. | GPL22945 | 12074 |
| GSM2811060 | H7-Tub-LD1184 | GSM2811060 | tissue: Tubulointerstitium from kidney biopsy |  | GeneChip arrays were scanned by GeneChip Scanner 3000 7G according to the Affymetrix Expression Analysis Technical Manual. | Gene expression from the tubulointerstitial compartment of human H7-Tub-LD1184 | Each batch and platform was processed individually with its corresponding BrainArray CDF and normalized by RMA. After individual batch and platform processing, samples were merged by common EntrezGeneIDs, and finally batch corrected using Combat. | GPL22945 | 12074 |
| GSM2810894 | H1-Tub-DN1 | GSM2810894 | tissue: Tubulointerstitium from kidney biopsy | diagnosis: Diabetic nephropathy | GeneChip arrays were scanned by GeneChip Scanner 3000 7G according to the Affymetrix Expression Analysis Technical Manual. | Gene expression from the tubulointerstitial compartment of human H1-Tub-DN1 | Each batch and platform was processed individually with its corresponding BrainArray CDF and normalized by RMA. After individual batch and platform processing, samples were merged by common EntrezGeneIDs, and finally batch corrected using Combat. | GPL24120 | 12074 |
| GSM2810895 | H1-Tub-DN10 | GSM2810895 | tissue: Tubulointerstitium from kidney biopsy | diagnosis: Diabetic nephropathy | GeneChip arrays were scanned by GeneChip Scanner 3000 7G according to the Affymetrix Expression Analysis Technical Manual. | Gene expression from the tubulointerstitial compartment of human H1-Tub-DN10 | Each batch and platform was processed individually with its corresponding BrainArray CDF and normalized by RMA. After individual batch and platform processing, samples were merged by common EntrezGeneIDs, and finally batch corrected using Combat. | GPL24120 | 12074 |
| GSM2810896 | H1-Tub-DN11 | GSM2810896 | tissue: Tubulointerstitium from kidney biopsy | diagnosis: Diabetic nephropathy | GeneChip arrays were scanned by GeneChip Scanner 3000 7G according to the Affymetrix Expression Analysis Technical Manual. | Gene expression from the tubulointerstitial compartment of human H1-Tub-DN11 | Each batch and platform was processed individually with its corresponding BrainArray CDF and normalized by RMA. After individual batch and platform processing, samples were merged by common EntrezGeneIDs, and finally batch corrected using Combat. | GPL24120 | 12074 |
| GSM2810897 | H1-Tub-DN2 | GSM2810897 | tissue: Tubulointerstitium from kidney biopsy | diagnosis: Diabetic nephropathy | GeneChip arrays were scanned by GeneChip Scanner 3000 7G according to the Affymetrix Expression Analysis Technical Manual. | Gene expression from the tubulointerstitial compartment of human H1-Tub-DN2 | Each batch and platform was processed individually with its corresponding BrainArray CDF and normalized by RMA. After individual batch and platform processing, samples were merged by common EntrezGeneIDs, and finally batch corrected using Combat. | GPL24120 | 12074 |
| GSM2810898 | H1-Tub-DN3 | GSM2810898 | tissue: Tubulointerstitium from kidney biopsy | diagnosis: Diabetic nephropathy | GeneChip arrays were scanned by GeneChip Scanner 3000 7G according to the Affymetrix Expression Analysis Technical Manual. | Gene expression from the tubulointerstitial compartment of human H1-Tub-DN3 | Each batch and platform was processed individually with its corresponding BrainArray CDF and normalized by RMA. After individual batch and platform processing, samples were merged by common EntrezGeneIDs, and finally batch corrected using Combat. | GPL24120 | 12074 |
| GSM2810899 | H1-Tub-DN5 | GSM2810899 | tissue: Tubulointerstitium from kidney biopsy | diagnosis: Diabetic nephropathy | GeneChip arrays were scanned by GeneChip Scanner 3000 7G according to the Affymetrix Expression Analysis Technical Manual. | Gene expression from the tubulointerstitial compartment of human H1-Tub-DN5 | Each batch and platform was processed individually with its corresponding BrainArray CDF and normalized by RMA. After individual batch and platform processing, samples were merged by common EntrezGeneIDs, and finally batch corrected using Combat. | GPL24120 | 12074 |
| GSM2810900 | H1-Tub-DN6 | GSM2810900 | tissue: Tubulointerstitium from kidney biopsy | diagnosis: Diabetic nephropathy | GeneChip arrays were scanned by GeneChip Scanner 3000 7G according to the Affymetrix Expression Analysis Technical Manual. | Gene expression from the tubulointerstitial compartment of human H1-Tub-DN6 | Each batch and platform was processed individually with its corresponding BrainArray CDF and normalized by RMA. After individual batch and platform processing, samples were merged by common EntrezGeneIDs, and finally batch corrected using Combat. | GPL24120 | 12074 |
| GSM2810901 | H1-Tub-DN7 | GSM2810901 | tissue: Tubulointerstitium from kidney biopsy | diagnosis: Diabetic nephropathy | GeneChip arrays were scanned by GeneChip Scanner 3000 7G according to the Affymetrix Expression Analysis Technical Manual. | Gene expression from the tubulointerstitial compartment of human H1-Tub-DN7 | Each batch and platform was processed individually with its corresponding BrainArray CDF and normalized by RMA. After individual batch and platform processing, samples were merged by common EntrezGeneIDs, and finally batch corrected using Combat. | GPL24120 | 12074 |
| GSM2810902 | H1-Tub-DN8 | GSM2810902 | tissue: Tubulointerstitium from kidney biopsy | diagnosis: Diabetic nephropathy | GeneChip arrays were scanned by GeneChip Scanner 3000 7G according to the Affymetrix Expression Analysis Technical Manual. | Gene expression from the tubulointerstitial compartment of human H1-Tub-DN8 | Each batch and platform was processed individually with its corresponding BrainArray CDF and normalized by RMA. After individual batch and platform processing, samples were merged by common EntrezGeneIDs, and finally batch corrected using Combat. | GPL24120 | 12074 |
| GSM2810903 | H1-Tub-DN9 | GSM2810903 | tissue: Tubulointerstitium from kidney biopsy | diagnosis: Diabetic nephropathy | GeneChip arrays were scanned by GeneChip Scanner 3000 7G according to the Affymetrix Expression Analysis Technical Manual. | Gene expression from the tubulointerstitial compartment of human H1-Tub-DN9 | Each batch and platform was processed individually with its corresponding BrainArray CDF and normalized by RMA. After individual batch and platform processing, samples were merged by common EntrezGeneIDs, and finally batch corrected using Combat. | GPL24120 | 12074 |
| GSM2811026 | H5-Tub-LD373 | GSM2811026 | tissue: Tubulointerstitium from kidney biopsy |  | GeneChip arrays were scanned by GeneChip Scanner 3000 7G according to the Affymetrix Expression Analysis Technical Manual. | Gene expression from the tubulointerstitial compartment of human H5-Tub-LD373 | Each batch and platform was processed individually with its corresponding BrainArray CDF and normalized by RMA. After individual batch and platform processing, samples were merged by common EntrezGeneIDs, and finally batch corrected using Combat. | GPL24120 | 12074 |
| GSM2811027 | H5-Tub-LD374 | GSM2811027 | tissue: Tubulointerstitium from kidney biopsy |  | GeneChip arrays were scanned by GeneChip Scanner 3000 7G according to the Affymetrix Expression Analysis Technical Manual. | Gene expression from the tubulointerstitial compartment of human H5-Tub-LD374 | Each batch and platform was processed individually with its corresponding BrainArray CDF and normalized by RMA. After individual batch and platform processing, samples were merged by common EntrezGeneIDs, and finally batch corrected using Combat. | GPL24120 | 12074 |
| GSM2811028 | H5-Tub-LD375 | GSM2811028 | tissue: Tubulointerstitium from kidney biopsy |  | GeneChip arrays were scanned by GeneChip Scanner 3000 7G according to the Affymetrix Expression Analysis Technical Manual. | Gene expression from the tubulointerstitial compartment of human H5-Tub-LD375 | Each batch and platform was processed individually with its corresponding BrainArray CDF and normalized by RMA. After individual batch and platform processing, samples were merged by common EntrezGeneIDs, and finally batch corrected using Combat. | GPL24120 | 12074 |

**Supplementary Table S1.** Detailed information about the final input samples of WGCNA

| ID | Description | GeneRatio | BgRatio | pvalue | p.adjust | qvalue | geneID | Count |
| --- | --- | --- | --- | --- | --- | --- | --- | --- |
| GO:1902175 | regulation of oxidative stress-induced intrinsic apoptotic signaling pathway | 2/49 | 27/18670 | 0.002271 | 0.089286 | 0.073787 | P4HB/SOD2 | 2 |
| GO:0033209 | tumor necrosis factor-mediated signaling pathway | 3/49 | 167/18670 | 0.009583 | 0.170689 | 0.141059 | PSME3/TNFRSF12A/ACTN4 | 3 |
| GO:0007220 | Notch receptor processing | 1/49 | 10/18670 | 0.025944 | 0.223983 | 0.185101 | PSENEN | 1 |

**Supplementary Table S2.** Detailed information about the functional enrichment of the dark orange module.

| ID | Description | GeneRatio | BgRatio | pvalue | p.adjust | qvalue | geneID | Count |
| --- | --- | --- | --- | --- | --- | --- | --- | --- |
| GO:0038066 | p38MAPK cascade | 3/49 | 53/18670 | 0.000363 | 0.022357 | 0.018037 | LGALS9/XDH/ZC3H12A | 3 |
| GO:0022407 | regulation of cell-cell adhesion | 6/49 | 403/18670 | 0.000622 | 0.030386 | 0.024514 | CD3E/CD4/LGALS9/CD70/CD40LG/ZC3H12A | 6 |
| GO:0001819 | positive regulation of cytokine production | 5/49 | 464/18670 | 0.007202 | 0.105206 | 0.084874 | CD3E/CD4/LGALS9/NFATC4/CD40LG | 5 |

**Supplementary Table S3.** Detailed information about the functional enrichment of the dark red module.

| ID | Description | GeneRatio | BgRatio | pvalue | p.adjust | qvalue | geneID | Count |
| --- | --- | --- | --- | --- | --- | --- | --- | --- |
| hsa04512 | ECM-receptor interaction | 3/32 | 88/8034 | 0.005007 | 0.189867 | 0.170407 | SPP1/ITGB3/ITGA3 | 3 |
| hsa04510 | Focal adhesion | 4/32 | 201/8034 | 0.0079 | 0.189867 | 0.170407 | SPP1/ITGB3/ITGA3/ACTN4 | 4 |
| hsa04151 | PI3K-Akt signaling pathway | 5/32 | 354/8034 | 0.012188 | 0.189867 | 0.170407 | SPP1/ITGB3/EIF4E2/ITGA3/GNB2 | 5 |

**Supplementary Table S4.** Detailed information about the significant enriched pathways of the dark orange module.

| ID | Description | GeneRatio | BgRatio | pvalue | p.adjust | qvalue | geneID | Count |
| --- | --- | --- | --- | --- | --- | --- | --- | --- |
| hsa04514 | Cell adhesion molecules (CAMs) | 3/22 | 148/8034 | 0.007297 | 0.138651 | 0.117143 | CD4/CD22/CD40LG | 3 |
| hsa04658 | Th1 and Th2 cell differentiation | 2/22 | 92/8034 | 0.025825 | 0.178426 | 0.150747 | CD3E/CD4 | 2 |
| hsa04060 | Cytokine-cytokine receptor interaction | 3/22 | 294/8034 | 0.044662 | 0.230992 | 0.19516 | CD4/CD70/CD40LG | 3 |

**Supplementary Table S5.** Detailed information about the significant enriched pathways of the dark red module.

| ID | Description | GeneRatio | BgRatio | pvalue | p.adjust | qvalue | geneID | Count |
| --- | --- | --- | --- | --- | --- | --- | --- | --- |
| GO:0030198 | extracellular matrix organization | 23/202 | 368/18670 | 0.0000000000037431 | 0.0000000141 | 0.0000000111 | NDNF/TNFRSF11B/MMP7/COL4A1/COL4A2/VCAN/VCAM1/PXDN/TGFBI/TIMP1/ITGB6/ANXA2/CTSS/COL3A1/LUM/COL15A1/CTSV/COL1A2/TNC/RGCC/COL6A3/POSTN/PLG | 23 |
| GO:0007162 | negative regulation of cell adhesion | 14/202 | 289/18670 | 0.00000331417 | 0.000194 | 0.000152 | DUSP1/PTPRO/PODXL/HRG/SERPINE2/TGFBI/FGL2/ANXA1/LGALS1/TNC/RGCC/ARHGDIB/POSTN/PLG | 14 |
| GO:0030449 | regulation of complement activation | 8/202 | 115/18670 | 0.0000314071 | 0.001142 | 0.000895 | CFB/CLU/C1S/C1R/C3/SERPING1/C1QA/C1QB | 8 |
| GO:0010631 | epithelial cell migration | 11/202 | 351/18670 | 0.001577 | 0.02277 | 0.017838 | FGF1/HRG/TAC1/NR4A1/PLK2/ANXA1/ANXA3/RGCC/APOH/SERPINF1/S100A2 | 11 |

**Supplementary Table S6.** Detailed information about the significant enriched terms of DEGs related to DN.

| ID | Description | GeneRatio | BgRatio | pvalue | p.adjust | qvalue | geneID | Count |
| --- | --- | --- | --- | --- | --- | --- | --- | --- |
| hsa04064 | NF-kappa B signaling pathway | 6/120 | 104/8034 | 0.004523 | 0.02763 | 0.02192 | GADD45A/GADD45B/VCAM1/LY96/LTB/CCL19 | 6 |
| hsa04512 | ECM-receptor interaction | 7/120 | 88/8034 | 0.000325 | 0.003666 | 0.002908 | COL4A1/COL4A2/THBS2/ITGB6/COL1A2/TNC/COL6A3 | 7 |
| hsa04514 | Cell adhesion molecules (CAMs) | 9/120 | 148/8034 | 0.000352 | 0.003764 | 0.002986 | VCAN/CLDN3/VCAM1/HLA-DPA1/HLA-DRA/HLA-DMA/HLA-DRB1/HLA-DQB1/HLA-DQA1 | 9 |
| hsa04151 | PI3K-Akt signaling pathway | 12/120 | 354/8034 | 0.006449 | 0.034454 | 0.027334 | FGF1/SGK1/G6PC/COL4A1/COL4A2/NR4A1/THBS2/ITGB6/EGF/COL1A2/TNC/COL6A3 | 12 |

**Supplementary Table S7.** Detailed information about the significant enriched pathways of DEGs related to DN.

| name | MNC | Degree | Closeness | Betweenness |
| --- | --- | --- | --- | --- |
| C3 | 34 | 35 | 96.75 | 1931.606 |
| PLG | 22 | 22 | 89.25 | 626.1214 |
| ANXA2 | 19 | 20 | 86.91667 | 1026.443 |
| FOS | 22 | 24 | 89.91667 | 2086.072 |
| ALB | 53 | 58 | 111.5833 | 8249.245 |
| EGF | 37 | 41 | 101.1667 | 3953.815 |
| TIMP1 | 39 | 39 | 100.1667 | 2344.047 |
| CCL2 | 34 | 35 | 98.16667 | 2919.148 |
| CLU | 22 | 23 | 88.66667 | 682.3268 |
| ANXA1 | 17 | 18 | 84.08333 | 804.8234 |
| VCAM1 | 28 | 28 | 93.33333 | 1987.336 |

**Supplementary Table S8.** Exact value of the Degree, Betweenness, Closeness and MNC for hub genes.
